# What causes cough in pulmonary fibrosis, and how should we treat it?

**DOI:** 10.1097/MCP.0000000000001087

**Published:** 2024-06-24

**Authors:** Katherine J. Myall, Peter S.P. Cho, Surinder S. Birring

**Affiliations:** a Department of Respiratory Medicine, King's College Hospital; b King's College London, London, UK

**Keywords:** chronic cough, cough hypersensitivity, interstitial lung disease

## Abstract

**Purpose of review:**

To review the current understanding of the impact, mechanisms and treatments for cough in patients with interstitial lung disease (ILD). Evidence suggests that cough is a prevalent symptom in patients with ILD and has a significant impact on patients.

**Recent findings:**

There is increasing interest in the role of cough hypersensitivity as seen in chronic refractory cough in patients with ILD, and encouraging recent results suggest that ILD-associated cough responds to opiate therapy.

**Summary:**

Understanding the aetiology of cough in patients with ILD is crucial to continue to develop therapies which might be effective in reducing cough and increasing quality of life.

## INTRODUCTION

Interstitial lung disease (ILD) is an umbrella term for a diverse group of diseases connected by the infiltration of lung parenchyma with extracellular matrix, leading to restrictive lung disease and respiratory failure over time. The archetypal ILD is idiopathic pulmonary fibrosis (IPF), accounting for around a third of all cases. However, irrespective of the initial presentation of ILD, a proportion patients will go on to develop a progressive phenotype with clinical, histopathological and radiological features mirroring those of IPF, and bearing a similar prognosis. Collectively these are termed progressive pulmonary fibrosis (PPF) [1,2].

Cough is a frequently occurring symptom for patients with ILD, may precede diagnosis, and besides dyspnoea is one of the most distressing symptoms. It is often described by patients as dry and hacking [3]. In a cross-sectional analysis, Lan *et al.* found that 81% of patients with IPF complained of cough, with patients with connective-tissue disease associated-ILD (CTD-ILD) having a cough prevalence of 66%; NSIP 71% and pulmonary sarcoidosis 70% [4]. However, using a cut-off score of 14 or less in the Leicester Cough Questionnaire (LCQ) – a validated cough-specific measure – in a cross sectional study of 1447 patients, Lee *et al.* found that only 24.8% of patients with ILD had a moderate-severe cough. Key *et al.* found that cough frequency in IPF was similar to that in patients with chronic refractory cough (CC-defined as an unexplained cough for a period of >8 weeks, persisting despite treatment, and frequency correlated negatively with quality of life as measured by the LCQ [5].

Multiple studies have found an association between disease severity and cough in patients with ILD. Genome-wide association studies have shown that the presence of a common polymorphism in the promoter region of MUC5B (rs35705950) is associated with the development of familial ILDs and IPF, and Scholand *et al.* demonstrated an association between expression of the mutant rs35705950 allele of MUC5B and cough severity, perhaps suggesting a direct link between the development of fibrosis and cough [6,7]. Whilst smaller studies have not been able to detect an association between cough and disease severity, Ryerson *et al.* in their study of 242 patients found an association between cough and severity of ILD as measured by forced vital capacity (FVC) and exertional desaturation. It should be noted that their study was limited by patients simply reporting cough as a symptom of their disease [8]. However, further work by Lan *et al.* in 179 patients demonstrated that cough severity and health status according to the visual analogue scale (VAS) and LCQ were associated with lower baseline FVC, gas transfer and walk distance on 6 min-walk test, as well as exertional desaturation [4]. Cough data was also embedded within the scleroderma lung study, and patients with cough had a lower baseline gas transfer, which improved with treatment of the ILD [9]. Contrary to these findings, the PROFILE study, a UK-based cross-sectional study of IPF patients found only a weak association between LCQ score and baseline FVC [10].

The presence of cough also associates with disease progression in some studies. Zaman *et al.* found that the presence of cough was negatively associated with transplant-free survival in men with IPF [11]. This finding was replicated by Lee *et al.* who demonstrated that LCQ predicted respiratory hospitalization, mortality and lung transplantation, and in an Australian registry study Jo *et al.* weak but significant association between cough and mortality [12,13]. More recent work by Khor *et al.* reported findings from 3886 participants in the Canadian Registry for Pulmonary Fibrosis who performed cough VAS at baseline and were followed up longitudinally. The authors found that cough severity both as a continuous variable and using a 30 mm cutoff were independently associated with greater decline in gas transfer, evidence of disease progression and worse transplant-free survival in both IPF and non-IPF ILD [14]. These studies all included heterogeneous populations of patients with ILD across multiple centres, and used validated cough patient reported outcome measures, but lacked objective measures of cough frequency. In contrast, recent data from the PROFILE study was not able to find an association between the LCQ and mortality in patients with IPF after correcting for baseline FVC [10].

Mortality data and measures of lung function have long been used to measure disease progression in ILD, but are imperfect measures, and to fully understand the association between cough and disease severity and disease progression in ILD, larger well designed prospective studies, including objective cough measurement along with validated cough-specific patient-reported outcome measures and direct measures of fibrosis with CT imaging are required. However, the presented data would appear to suggest that the fibrotic lung may be associated with cough, and that as the disease progresses with reduced lung volumes and diffusion capacity, cough becomes more prevalent and severe. Despite this, the pathobiology of cough in ILD is poorly understood, with proposed mechanisms including mechanical and inflammatory stimulation of mechanoreceptors and chemoreceptors, as well as cough hypersensitivity. 

**Box 1:**
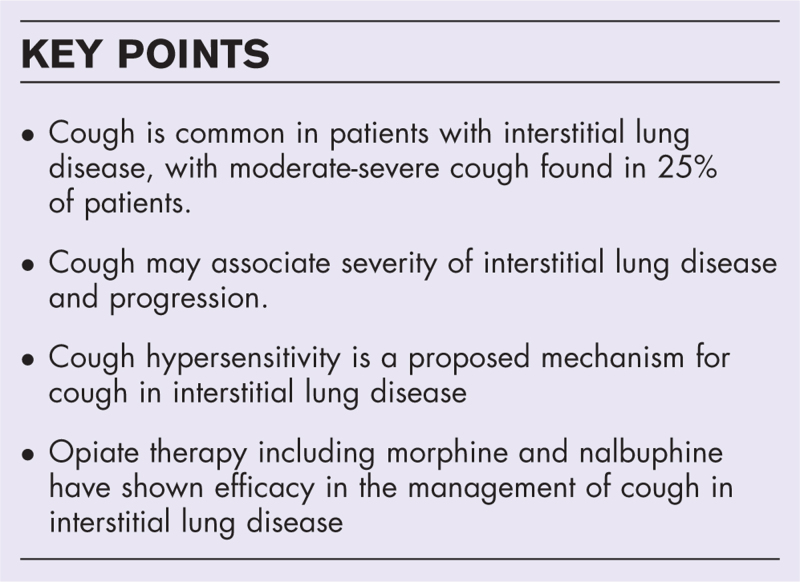
no caption available

## WHAT CAUSES COUGH IN PULMONARY FIBROSIS?

### Cough reflex

Cough is a physiological defensive response to irritations to the airways, and the cough reflex can be diminished or heightened in diseases and lead to pathological cough.

Pulmonary afferent sensory neurones can be categorised by their conduction speeds to myelinated Aδ-fibres and unmyelinated C-fibres [15]. Aδ-fibres include the rapidly adapting receptors (RAR), and *in vitro* observations suggest that Aδ-fibres respond to mechanical stimulations and hypertonic saline, but not chemical stimuli. [16]. C-fibres, on the other hand, are polymodal and are sensitive to both chemical and mechanical stimuli, including capsaicin and bradykinin [16]. Interestingly, Aδ-fibres do not appear to obey the selectivity with stimuli described above *in* vivo, and appear to respond to chemical stimuli. It is believed that such Aδ-fibre activations are in fact secondary to the effects of C-fibres activation *in vivo*. Taken together, multiple receptors and sensory afferent neurones are likely responsible for the mechanisms of cough through intricate interactions. These sensory neurons travel via the vagus nerve to the brainstem and stimulate the release of neuropeptides such as substance P and neurokinin which in turn act on neurons within the brainstem which trigger cough via efferent nerves within the vagus, phrenic and spinal nerves [17]. The pivotal role of the vagus nerve in the sensory pathway of cough is highlighted by the fact that cough is abolished by bilateral vagotomy in both animals and humans [18,19].

The central cough mechanisms remain largely enigmatic. Recently, functional neuroimaging had yielded novel insight to the central cough neural pathways for urge-related cough (anterior insula and primary sensory cortex) and voluntary suppression of cough (dorsomedial prefrontal cortex and right inferior frontal gyrus) [20–22].

### Cough hypersensitivity

In chronic cough (CC), recent evidence suggests that neurogenic or neuropathic mechanisms may be responsible. The symptom profile is akin to that of chronic pain, with increased response to tussive (hypertussia) and nontussive (allotussia) stimuli. Biopsies from the airway of patients with CC suggest that nerve length and branching points within the epithelium are increased, suggesting neural hyperplasticity [23]. Functional magnetic resonance imaging also suggests that there are functional differences within the brain of people with CC compared with healthy controls, which correlate with capsaicin sensitivity [24]. Patients with CC also exhibit an impaired ability to suppress cough in response to tussive stimuli, and cough responds to treatments aimed at peripheral and central nerves including Gefapixtant (P2X3 inhibitor), gabapentin, and morphine, as well as cough suppression speech therapy [25–29].

**FIGURE 1:**
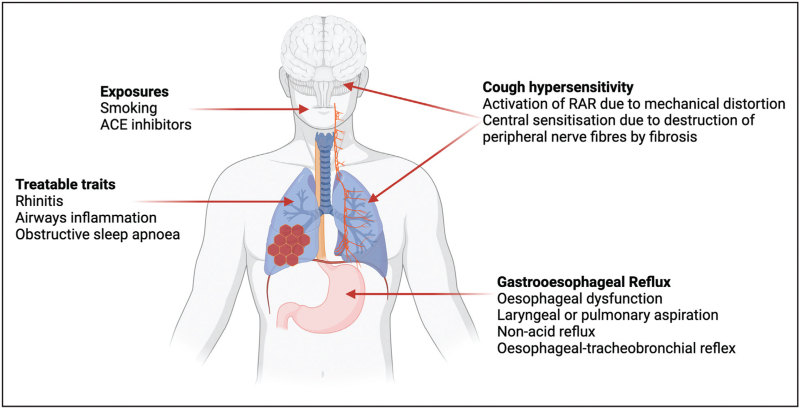
Proposed mechanisms for cough in interstitial lung disease may be categorised into exposures; treatable traits; cough hypersensitivity and gastro-oesophageal reflux.

In patients with ILD, several studies have demonstrated hypersensitivity to tussive stimuli such as capsaicin or substance P [30,31]. Neurotrophins including nerve growth factor (NGF) and brain-derived neurotrophic factor (BDNF) are found at increased levels in the sputum of patients with IPF when compared to healthy controls and may play a role in hypersensitivity to tussive stimuli [31]. Patients with ILD exhibit features of cough hypersensitivity including allotussia, hypertussia and laryngeal paraesthesia [32]. We discuss several potential mechanisms which might drive cough hypersensitivity in patients with ILD here (Fig. 1), although it should be noted that it is by no means certain that this is the mechanism by which patients with ILD develop chronic cough.

### Mechanical stress as a cause of cough in pulmonary fibrosis

As discussed, self-reported cough is associated with greater volume restriction in patients with IPF, and cough sensitivity was increased in patients challenged with capsaicin when compared to healthy controls [4,8,30]. However, this effect was not reproduced by extra-thoracic compression mimicking reduced lung volumes, suggesting that volume reduction alone is not sufficient to induce cough. In IPF, the pathognomonic histopathological and radiological finding is of usual interstitial pneumonia, in which fibrotic lesions are disseminated in time and space. Airways adjacent to fibrotic lung are distorted and it has been hypothesised that this peribronchial fibrosis might lead to increased stimulation of RAR sensory afferents [33]. A study by Jones *et al.* lends weight to this hypothesis by examining the effect of external percussion on cough in patients with IPF [34]. Percussion stimulation triggered cough in 23 of 27 patients with IPF compared with just 5 of 30 healthy controls (*P* < 0.001). Mean cough counts were higher at all sites of stimulation but required lower frequency stimulation at the posterior lung base compared to the anterior upper chest and manubrium sternum. Since this is where fibrosis is typically most severe in patients with IPF, the authors hypothesized that the external vibratory stimulus was more readily transmitted through the fibrotic lung to RARs situated within the airway.

### Airway clearance and mucous production

Whilst the cough typically described by patients with ILD is typically dry, in patients with tractional airways dilatation causing bronchiectasis, the cough may be productive [17]. As discussed, the common MUC5B promoter polymorphism predisposes to ILD. MUC5B codes for the predominant mucin within the distal airway with a role in mucociliary clearance and co-locates with the honeycomb cysts found in IPF [35,36]. Mucins are also important in the development of non-CF bronchiectasis, and together with the findings of Scholand *et al.* that cough-specific health status LCQ is increased in patients expressing the minor T-allele, this might suggest a role for mucous impaction [7]. However, there is limited evidence from clinical practice that traction bronchiectasis associates with cough, beyond a small retrospective study of 23 patients with IPF in whom those with self-reported cough (10/23; 43%) had radiographic evidence of traction bronchiectasis in 83.3% compared with 11.7% in those with no cough (*P* < 0.01) [37].

### Co-morbid causes of cough in pulmonary fibrosis

Patients with ILD are commonly affected by other conditions predisposing to cough including smoking, gastro-oesophageal reflux disease (GERD), chronic obstructive pulmonary disease (COPD), asthma, rhinitis, and obstructive sleep apnoea (OSA). Patients with hypertension are also frequently prescribed angiotensin-converting enzyme (ACE inhibitors), a well recognized cause of cough. Indeed, in their study, Madison *et al.* reported that a comorbid cause for cough was found in over 50% of ILD patients referred for evaluation of cough. This population may not be representative of the ILD population as a whole, and Lan *et al.* in their large cross-sectional study found comorbid conditions in patients with and without cough [4,38].

#### Gastro-oesophageal reflux disease

GERD is strongly associated with both chronic cough and ILD and is postulated as the cause of chronic cough in ILD. It is therefore tempting to suppose that GERD might be a cause of cough in patients with ILD via mechanisms, which might include laryngeal or pulmonary aspiration; the oesophageal–tracheobronchial reflex – in which shared vagal innervation converging in the brainstem and cortical processing centres leads to potential cross-stimulation; and airway reflux secondary to oesophageal dysfunction caused by distortion by the fibrotic lung. In the Canadian registry study, cough severity was independently associated with GERD in both IPF and non-IPF ILD [14]. In IPF, 73% of patients have ineffective oesophageal motility (IEM) [39], which associates with a lower FVC, and there is evidence of acid reflux even in asymptomatic patients [40]. Oesophageal pathology may be yet more significant in other causes of ILD, for example in patients with scleroderma, which is associated with both oesophageal pathology and ILD [41]. Whilst early studies suggested that treatment of GERD with either proton pump inhibitors or fundoplication might be effective, a more recent meta-analysis was not able to demonstrate a benefit of on quality of life, progression-free survival or all-cause mortality [42]. This concurs with a study by Kilduf *et al.* in which treatment of acid reflux with high-dose proton pump inhibitors in patients with IPF was not associated with a reduction in cough count in patients with IPF, and nonacid reflux events were actually increased [43]. This finding led to consideration of nonacid reflux as a cause of cough in ILD patients. In chronic cough patients, there is no difference in cough thresholds between patients with acid and nonacid reflux [44]. Weakly-acidic reflux events are increased in IPF patients, and bile acid and pepsin are seen at higher rates in the saliva and bronchoalveolar fluid of patients with IPF and other ILD than in healthy controls indicating aspiration of gastric contents [43].

#### Obstructive sleep apnoea

OSA is common in patients with fibrotic interstitial lung disease, with a prevalence of at least moderate OSA of around 1/3 even in patients who have no daytime symptoms of sleepiness [45]. OSA is frequently associated with a chronic cough, which may be the presenting feature, and responds to CPAP therapy [46].

## HOW SHOULD WE TREAT COUGH IN PULMONARY FIBROSIS?

### Management of co-morbid conditions

The current guidance suggests that the management of cough in ILD should include smoking cessation and the discontinuation of angiotensin converting enzyme (ACE) inhibitors [47]. Clearly, where co-morbid conditions exist which may be the cause of the cough, optimal management of these is recommended. However, treatment of GERD is suggested only if there is symptomatic acid reflux [47]. The TIPAL study is currently recruiting IPF patients to a randomized control study of PPI in patients without symptomatic reflux. This study includes objective cough frequency as a primary outcome measure. There is limited data from observational studies on the efficacy of pro-kinetics such as domperidone or metoclopramide in this population and caution is advised given the significant adverse effects. Nonpharmacological measures such as lifestyle and dietary advice (weight loss; eating earlier; avoidance of alcohol and caffeine), raising the head of the bed and wearing loose clothing are likely to be of significant benefit. Given the lack of data at present, we would not support fundoplication for the management of ILD-associated cough. Given the emerging evidence for cough hypersensitivity, the guidelines also suggest referring to the CC guideline including the use of neuromodulators such as gabapentin in patients with refractory cough [47]. To our knowledge, there is no evidence to support treatment of traction bronchiectasis in IPF with therapies traditionally used to treat patients with bronchiectasis from other causes such as hypertonic saline, and physiotherapy techniques to aid clearance of sputum. However, this may be considered in cases where there is standalone bronchiectasis.

### Novel therapies for cough in interstitial lung disease

#### Treatment of interstitial lung disease

In IPF, there are two available antifibrotic medications, pirfenidone and nintedanib, which slow the progression of disease as measured by FVC decline. However, the trials did not improve quality of life and failed to include validated cough outcome measures [48,49]. Posthoc analysis of an early trial of pirfenidone did not suggest improvement in the cough-specific domains of a composite symptom score following treatment with pirfenidone [50]. However, a study by van Manen *et al.* demonstrated a 34% improvement in cough frequency following pirfenidone initiation in 43 patients with IPF, with simultaneous improvement in cough VAS and LCQ scores [51]. This may simply represent regression to the mean given that patients had severe cough at initiation, and in a study of nebulized pirfenidone, objective cough frequency and LCQ were prospectively studied, and there was no treatment effect [52]. In PPF, there was no effect of pirfenidone on cough severity as measured by LCQ, although it must be noted that this study did not meet its primary endpoint of change in FVC as measured by home spirometry [53]. The INBUILD trial of nintedanib in PPF also did not include cough-specific outcome measures, but there was a small but significant effect on the rate of decline in the cough domain of the L-PF questionnaire in patients treated with Nintedanib [54].

#### Antitussive therapies

Until recently, there was limited evidence for the use of antitussive therapies in ILD, with evidence limited to small-scale proof of concept studies. Lutherer *et al.* trialled oral interferon-α in 12 patients, 6 of whom had chronic cough, and of these, 5 had improvement in LCQ and cough VAS with treatment [55]. However, this uncontrolled trial has not since been repeated in a larger cohort of patients.

The use of thalidomide for its anti-inflammatory and peripheral nerve effects in a double-blind placebo-controlled crossover trial of 24 patients with IPF and chronic cough resulted in a reduction of 11.4 points in the primary outcome measure, the cough quality of life questionnaire, as well as improved cough VAS [56]. This change was statistically significant although did not meet the minimal clinically important difference of 13 points determined in a CC population. However the drug has not been routinely adopted in clinical practice due to cost implications and side effect profile.

Nebulized sodium chromoglycate decreases inflammation and sensory c-fibre activation and was therefore proposed as a treatment for chronic cough in IPF. Birring *et al.* performed a multicentre double-blind trial of the drug in patients with IPF-associated cough [57].There was a 30% reduction in objective cough frequency with treatment, and a trend towards improved cough-related quality of life, although the study was underpowered to detect this. This treatment had previously been ineffective in a CC population, with this treatment difference was argued to suggest that mechanism in IPF-cough differed from that of chronic cough. However, the follow-up SCENIC trial in IPF patients also failed to replicate the treatment effect [58]. This study was curtailed by the COVID-19 pandemic and recruited only 108 of a planned 180 participants, but failed to meet its primary endpoint of reduction in objective cough frequency. The authors speculate that this was due to voluntary control of cough making studies vulnerable to placebo effect.

The antitussive agent Gefapixant has efficacy in CC by binding the P2X3 receptor on C-fibres [59]. However, in a randomized placebo-controlled crossover trial of Gefapixant in 51 patients with IPF-associated cough, Martinez *et al.* found no significant effect of Gefapixant on awake cough frequency [26]. This study was affected by methodological issues including small sample size, protocol deviation and skewed cough frequency which might affect the interpretation of the findings. However, it met all of its secondary endpoints including validated patient-reported outcome measures and a re-analysis of the data using a logarithmic scale to account for the skewed data yielded positive results.

More recently, there has been interest in the use of opiates for the management of cough in IPF. Morphine has demonstrated efficacy in the treatment of CC, as morphine is thought to act centrally within the brainstem to suppress the cough reflex [28]. In a trial of modified-release morphine at 5 mg b.i.d. in a phase 2 multicentre placebo-controlled two-way crossover trial in 47 patients with IPF and a self-reported chronic cough, objective cough count was reduced by 39.4% in treated patients with no change in the placebo arm [60^▪▪^]. Nausea and constipation occurred in 14% and 21% respectively, with 98% of patients completing treatment. Nalbuphine (an opiate agonist-antagonist) has also been trialled, with the hypothesis that patients might experience fewer side-effects and a reduced risk of dependency. Maher *et al.* demonstrated in a randomized double-blind placebo-controlled crossover trial of 41 patients with IPF and cough, that objective daytime cough frequency was reduced by 52.5% placebo-adjusted decrease from baseline, with associated cough-specific improvements in quality of life [61^▪▪^]. However, treatment-associated adverse effects including nausea, fatigue, constipation and dizziness were seen in 39%, 37%, 32% and 42%, respectively. It is significant that the authors observed the expected placebo effect in this study, as compared to the morphine study. Together, these positive studies may lend support to the cough hypersensitivity hypothesis as a mechanism of cough in ILD, and also offer potential treatments for IPF-associated cough, which are currently lacking.

Other agents in trials of IPF-cough at present include orvepitant – an NK1 receptor antagonist which blocks substance P, and which has had demonstrated efficacy in managing cough in lung cancer patients [62].

## CONCLUSION

Cough is a frequently-occurring and often debilitating symptom for patients with ILD. However, the underlying mechanisms remain incompletely understood, and mechanistic studies, particularly into the role of cough hypersensitivity remain crucial. It remains unclear if treatment of fibrosis with the currently available antifibrotic therapies has an effect on cough; however given that they slow the rate of disease rather than resolve existing fibrosis, it is likely that any effect will be small and simply slow worsening rather than improve quality of life. The role of immunomodulatory and anti-inflammatory medications in the management of inflammatory ILD with associated cough remains unexplored. Therefore, the role of antitussives in improving quality of life remains a priority. The recent positive trials of opiate therapy are extremely encouraging, but require larger-scale studies in IPF and diseases other than IPF. Further trials of P2X3 are warranted given the methodological issues in the existing data. Trials of nonpharmacological measures effective in the management of CC of other causes such as speech and language therapy and physiotherapy are in development.

## Acknowledgements

*None.*

### Financial support and sponsorship

*None.*

### Conflicts of interest

*S.B.: Consultancy: personal fees from Nerre, Trevi and Merck.*

## REFERENCES AND RECOMMENDED READING

Papers of particular interest, published within the annual period of review, have been highlighted as:

▪ of special interest
▪▪ of outstanding interest
